# Therapeutic work with clients living in poverty

**DOI:** 10.1177/00207640221139798

**Published:** 2023-01-06

**Authors:** Elvera Ballo, Rachel Tribe

**Affiliations:** 1 EDB Consultancy, London, UK; 2 UEL and Queen Mary, University of London, UK

**Keywords:** Poverty-aware, therapy, IPA, mental health

## Abstract

**Background:**

Financial inequalities appear to be increasing and poverty is becoming ubiquitous. Poverty affects mental health but its impact on mental health and wellbeing is rarely highlighted within health research.

**Aims:**

The Covid-19 pandemic, the Ukrainian invasion and other international and national events have led to a cost-of-living crisis for many people. This is likely to lead to an increase in related referrals and therefore active consideration of the relevant issues relating to poverty appears vital. This paper reports a study which sought to understand how therapists experienced their work with clients who self-refer due to living in poverty.

**Method:**

Eight therapists participated in semi-structured interviews analysed using Interpretative Phenomenological Analysis (IPA).

**Results:**

Three superordinate themes were elicited: firstly ‘Resilience in the struggle to engage with therapeutic work’, secondly ‘Struggling to promote social activism’ and thirdly, ‘Navigating multiple challenges and barriers’. Each superordinate theme contains two or three sub themes.

**Conclusions:**

Issues of structural inequality (including but not limited to poverty) impact significantly on people’s lives but are often ignored or minimised in therapeutic work. It is important that therapists are aware of poverty and take this into account when working with clients.

## Introduction

Poverty, related distress and mental health are frequently found to be linked (Boardman et al., 2015; Elliott, 2016; Santiago et al., 2013). The potential for poverty to be detrimental to mental health and wellbeing appears clear (Elliott, 2016). Social justice issues such as poverty require attention by clinicians, as if left unacknowledged, they may see clients ‘trapped in social forces beyond their making’ (Kagan, 2015, p. 19). As Byrne and James (2020, p. 188) noted ‘We cannot ignore poverty as a predisposing, precipitating and maintaining factor in most of our patients’ disorders’. There is no doubt that poverty represents a meaningful aspect of clients’ distress and requires professional attention. Knifton and Inglis (2020) argue that poverty-aware practice requires embedding in mental health policy and practice and that this should include advocacy, commissioning and training. The importance of social justice issues (Tribe & Bell, 2018) and consideration of the social determinants of health are also being increasingly recognised, including in relation to mental health (Elliott, 2016; Weale, 2019).

The scientist practitioner and advocacy model (Mallinckrodt et al., 2015) which promotes the role of advocacy and foregrounds social justice issues in therapeutic work is one model that incorporates advocacy as a key component of mental health work. Although mental health services have often focussed on diagnostic categorisation and prescribing medication without giving adequate consideration to the context of people’s lives , although writing in relation to culture, the criticism might also be applied when considering people living in poverty (Marshall, 2022). Shame and stigma are frequently associated with living in poverty, this in addition to negative attitudes being held towards poverty may lead to prejudice and negative stereotyping by institutions, including those who deliver services (Shildrick & Russell, 2015). This may also influence help seeking behaviour. Chase and Walker (2013) argue that therapeutic services need to incorporate consideration of the potential impact of societal issues included poverty-driven stigma and shame into their work

## Poverty in the UK

In 2021 to 2022, 2.1 million emergency food parcels were given out by the Trussell Trust food banks in Britain, this is a rise of approximately 14% compared to 2019/20 (Trussell Trust, 2022). Whilst various measures have been used to define poverty, the figures given are normally relative to the median income for that year. In the UK for the period 2020/2021, approximately one in six people were reported to be on a relatively low income before housing costs, these numbers rose to approximately one in five when housing costs were included. This translates to 10.5 million people (16%) living on relative low incomes before housing costs and 13.4 million after housing costs (20%). 2.8 million children (19%) are included within this figures and after housing costs, the figure rises to 3.9 million children (27%). (House of Commons Library, 2022). As well as affecting health, the experience of poverty may lead to other hardships, for example homelessness (Goodman et al., 1991). Lister (2021) describes how living in poverty can be associated with people experiencing feelings of insecurity, powerlessness, a lack of agency and a loss of dignity and respect. All factors that might be detrimental to mental health. The Joseph Rowntree Foundation (2022) notes that inflation, rising energy, housing and other costs may push more people in the UK into poverty. Poverty can also lead to an inability to make long term plans, to developing a sense of security (McGrath et al., 2015) and to feelings of social exclusion (Weale, 2019). Child and pensioner poverty is on the increase with certain ethnic groups being disproportionally affected (Joseph Rowntree Foundation (2022).

## Therapists’ experience of working with clients living in poverty

In the UK, little has been published concerning psychological therapists’ responses in relation to their work with clients living in poverty and the complex nature of the poverty-mental health-therapeutic practice overlap. Notable exceptions include Banerjee and Bhattacharya (2022) who have written about the intersections of health inequality, poverty and psychological well being and Bhugra (1997), who wrote on this topic much earlier.

Therefore, by exploring the US literature on therapists who provide therapy with clients living in poverty, better insight into their practice may be obtained. Also, this may lead to an improvement in practice, training, and guidelines pertaining to interventions concerning people experiencing psychological distress as a result of living in poverty. Despite the association between mental health difficulties and the distressing circumstances of people living in poverty, there are few reviews on what kind of support needs to be offered and how prepared therapists are who work with clients presenting as living in poverty.

Smith et al. (2013) working in the USA reported that whilst therapists develop strong empathy for their clients who are living in poverty and maintained increased empathy, understanding and respect towards the plight of their clients, they also felt ‘overwhelmed’, ‘burned out’, and ‘overloaded’. Furthermore, these therapists attributed poverty as being a determining factor in their clients’ circumstances and alluded to systems that tend to trap clients into poverty. Smith et al. (2013) found that building a sound therapeutic relationship together with evidence-based treatment which consisted of intensive outreach, cultural sensitivity, and teamwork could lead to positive outcomes. Considering poverty related stressors and other barriers can present additional tasks for therapists who work with clients presenting with issues relating to living in poverty (Smith et al., 2013). They may have received little training on working with this structural inequality and the associated issues. Therapists’ may feel unprepared for this and some may experience stress in relation to this task (Smith, 2010).

Borges and Goodman (2020) highlights potential challenges inherent in working with clients living in poverty. For example, they highlight the powerlessness that clients living in poverty could be experiencing. Goodman et al. (2013) discovered that for some clients living in poverty, the therapeutic encounter may feel similar to those found in social services or welfare settings. Therefore, addressing the unequal balance of power dynamics in the relationship, negotiating and modifying boundaries within the therapeutic relationship was found to be key whilst exploring poverty-related stressors and contexts (Borges & Goodman, 2020).

## Study

Lack of research on how therapists work with clients who are living in poverty appears to be an important issue and statistical data infers that this is likely to be of increasing concern for clinicians. This study aimed to consider how therapists experience their work with clients who describe themselves as living in poverty. It was hoped this would increase awareness and understanding of such work and contribute to our understanding of these issues.

## Sample

Eight participants were recruited through ‘snowballing’ via NHS services, charities and private practices. Of the eight therapists, one described themself as Black British six as White British and one as British Asian. Two were aged between 40 and 49, three were 50 and 59, and three were 60 to 69 years of age. Four were trained as psychotherapists and four as psychologists, four defined themselves as male and four as female. All were senior clinicians, with three participants having 20 or more years post qualification experience and five having between 15 and 18 years. Ethical approval was obtained from the School of Psychology, UEL ethics committee.

## Method

The interviews were audio recorded and transcribed verbatim and analysed in line with Interpretative Phenomenological Analysis (IPA). The six-step IPA analytical process was used, (Smith et al., 2009). Superordinate themes were identified for each cluster. This process was revisited repeatedly, and themes reorganised until a clearer formation of the themes and sub-themes surfaced.

## Findings

Three superordinate themes provide an account of therapists’ experiences of offering psychotherapy to clients living in poverty: (1) Resilience and positioning the struggle to engage with this therapeutic work, (2) Struggling to promote social activism, (3) Navigating multiple challenges and barriers. Each sub theme contains two to three sub-themes which are detailed after the superordinate theme

## Superordinate theme: Resilience and positioning the struggle to engage with therapeutic work

All participants described resilience and perseverance in their therapeutic engagement with their clients who were living in poverty. They also cited how important they felt the work was despite the challenges, with many of them reflecting on their personal positioning in relation to this.

‘It’s important to try and to empower people to help me build up a resilience. That probably comes partly from my need to nurture, to help people to grow’

(Kate: 249–252).

This consideration of personal values can also be seen from Rose’s extract below.

‘This job was not just to be able to feed my children, but to do something I felt was worthwhile, that I had the capacity to do. . .and a sense of humanity. . .’ (Rose: 1015–1017).

Rose’s assertion reflected her personal experience of why she does this work. Kate’s commitment to helping her clients negotiate a system and a social world that contributes to their distress is detailed below.

‘I instil hope. . .they go from that unemployment to maybe working as a volunteer, 16 or 17 hours. They do not lose their money. I liaise with the job centres and get them to do maybe say a three-month course in computing or things like that’. (Kate: 222–229).

Kate also spoke of a desire to help her clients increase their social capital and opportunities through undertaking work which might not usually be seen to be within the remit of the clinician, for example liaising with other agencies. Whether clinicians should undertake this work when there is nobody to refer on to do this is a complex and contested issue which raises a number of opinions.

Resilience, commitment and a desire to help their clients as well as reflections on their own reasons for doing this work were all mentioned. Three main sub-themes from the data highlight this superordinate theme:

1. Commitment is affected by the circular process of the work
2. Therapists maintaining awareness of their own biases
3. Awareness of professional boundaries in the relationship

## Subtheme 1 – Commitment is affected by the circular process of the work

Many of the participants expressed a commitment to working with clients living in poverty in order to help prevent the vicious cycles which can perpetuate their client’s circumstances. Participants also expressed feeling disempowered given that they needed to rely on other agencies or workers including government agencies, to reinstate welfare benefits or other support services in alleviating their clients’ immediate poverty related distress. Participants also noted the lack of support for clients elsewhere should the participants decide to end therapy.

‘I can’t make this right, this feeling stuck. . .it’s certainly a deficit and needs more than supporting the individual and riding the circle until somehow they may manage to jump out of it’. (Kate: 459–464).

Kate’s use of the term ‘riding the circle’ implies going around and round with little hope of affecting their circumstances.

Liv explains what underlies her commitment to offering support to her clients is helping them see that they may have more choices than they think. However, the resources she was hoping to gain in her role as a therapist seem to mirror the meagre choices available to her clients living in poverty:

‘Poverty cuts down choices. . .hugely cuts down your choices especially when you are stuck and trapped, people compare themselves to others. With counselling it’s not that I want to move a client to a situation where you’re definitely going to do this or that, I want them to have more choices’. (Liv: 816–824).

## Sub theme 2 – Therapists maintaining awareness of their own biases

Cathy expresses her commitment to her work; she describes her own positioning but reflects on that of other therapists who see the issue of poverty as not relevant to their work. Thus, the biases and decisions of individual therapists may become highly influential in the way they work and the issues they prioritise.

‘I think that poverty has a direct effect on people’s lives, and colleagues’ feel that it’s not counselling to ever suggest any practical suggestion.’ (Cathy 408–411).

Jon is very careful in his use of language and seems to be aware of the role of labelling. Jon feels his work with this group of clients is to help them feel more dignified as human beings.

‘I don’t see destitute people. . . I see people on low income who work but with difficulty finding the bus fare to attend. . . I’m there to do that piece of work so people are less categorised and more fluid’ (Jon: 206–211).

Jon’s determination to help build a sense of value in his clients was the focus and what he saw as the major benefit of his therapeutic interventions and which reflected his own beliefs.

## Sub theme 3 – Awareness of professional boundaries in the relationship

Cathy conveys her sadness over the stigma facing some clients living with poverty even within the therapeutic profession.

‘.. it’s very sad when some of my colleagues who work in IAPT say that as soon as clients bring the “I’m broke” words, “I can’t pay rent or can’t eat”, they don’t have goals and so they are discharged or excluded’. (Cathy: 277–280).

Cathy’s comments reflected one of the profound difficulties for people living in poverty, that telling the truth about their difficult circumstances was interpreted within a particular IAPT service (and possibly more widely) as these service users being too lacking in motivation to be deemed to be ‘worthwhile’ clients. She appeared to express a sense of outrage at this boundary being brought in which further disadvantaged people who are already suffering from discrimination, alienation and desperation in their everyday lives.

For therapists who have a relatively affluent lifestyle, facing the issues that clients living with poverty face can challenge their ‘normal’ professional boundaries in a variety of ways. Kate described feeling tempted to push her professional boundaries in an effort to try and protect them from their struggles.

‘I think the bit I struggle with.. it feels quite powerful sometimes to want to be able to fix and you can’t, that’s frustration again. . . there’s something else I feel that I can’t quite name’. (Kate:174–183).

Setting boundaries, making the time itself valuable is a struggle for the therapist who can feel overwhelmed by their client’s isolation and struggles. Cathy articulates this struggle in the extract below:

‘But it’s very sad when you have a client with children and no help. We sometimes feel unable to help them move out within those 50 minutes. . .but we try and keep those boundaries. . .’

Sam discusses his experiences of dealing with absences and how this relates to boundaries, but the reason may not be of their making and how in some services, clients are penalised for this, in that the session is still counted as having been taken or may lead to them being discharged, but for clients living in poverty this can be for a good reason.

‘sometimes they don’t turn up.. or they cancel because they can’t afford to pay transport costs . . .they’ve fessed up and said, “yes, it was because I couldn’t afford to come”’. (laughs) (Sam: 104–109).

## Superordinate theme 2: Struggling to promote social activism

This superordinate theme emerged in the form of therapists’ advocacy on behalf of their clients by suggesting ways to try and move out of poverty in manageable steps, thus helping in the building of an effective ‘lifeline’ out of poverty, many of the therapists described a struggle to encourage this ‘action-oriented’ process. Three main sub-themes further highlight this superordinate theme:

1. Advocacy versus Therapy: Empowering versus disempowering
2. Difficulty engaging versus limited resources
3. Isolation versus Reaching Out

## Struggling to promote social activism

Social activism, in the form of advocating and the provision of information were seen as providing additional ways to assist clients. It was also seen as a way of informing other people about the impact of living in poverty on people’s lives. This gave rise to participants feeling a need to ‘take matters into their own hands’. Consequently, these efforts appeared to leave the participants on occasions in a position of not knowing whether their work was ethical, helpful or was supported within mainstream theoretical parameters.

‘In our clinics, we advocate all the time. . .its action. . .action. . .action outside the room. . .to help clients out of poverty and to empower otherwise we become stuck. . .then after a time they are beginning to work quite independently . . . it takes time. . .and. . …it becomes a lifeline.’ (Cathy: 100–106).

With tension in her voice, Cathy describes her feelings of frustration around the lack support and resources for her clients:

‘I feel overpowered and overwhelmed and wonder how clients cope from food banks for food, to second-hand shops for clothes, feeling like an under-class that nobody wants to associate with, that’s frightening so they really do need my help to act for them’. (Sam: 333–338).

Sam powerfully describes the limitations imposed on his clients by their experience of living in poverty. He acknowledges how frightening it is to be regarded as second-class citizens facing stigmatisation whilst they struggle to make ends meet, whilst trying to access the limited services that are out there to support them.

## Sub theme advocacy versus therapy: Empowering versus disempowering

Cathy describes the problems she faces in trying to advocate on behalf of her clients in dealing with services where she feels that their needs are not properly understood or adequately addressed:

‘I interact with the . . . bodies that are out there. . .it’s soul defeating. So, whether it’s going to be advocates for housing or for people to get the right kinds of benefit etc. . . they sometimes revert to things like taking drugs, drinking alcohol, so that their physiological problems are also increased. Sanctions don’t allow them to function at all. . .for me. . . .it’s not always about the money. . .it’s about giving back. . . that passion to act’. (Cathy: 109–116).

Cathy’s active participation in trying to help her clients access adequate housing and benefits raises other important issues in relation to people who live in poverty. For people who have learned to cope with poverty through drug and alcohol abuse, breaking those habits is challenging. Cathy’s trust and belief in the power of advocating seems unwavering as she powerfully expresses her desire to empower from within so that clients can learn to advocate themselves. This is clearly a struggle, and Cathy’s assertion that ‘it’s soul defeating’ raises the question of whether this was equally true for many of her clients.

‘I often say, “you’ve got a different background from me, I may misunderstand things, I may be saying something that’s a bit crass, I hope you’ll put me right.” I know there’s the other line of why should people from different groups always have to be educating everybody. . . you can’t learn about everybody’s culture in the world. . . and clients want me to ave these conversations . . . it’s not just about who gets what treatment but an opportunity to develop and to empower and accepting limitations.’ (Liv: 731–736).

Liv shows how she is trying to be truthful and open with her clients by acknowledging their differences, and respecting her responsibility to help her clients by openly addressing issues of diversity and power dynamics

## Sub-theme– Difficulty engaging versus limited resources

Leo provides an account of how uneasy he feels with regard to the systems that are in place and that resources are limited and for those who are less fortunate than he is and in need of help from the government:

‘I’m not quite sure, but I’m quite worried, fearful and a bit angry with the shortages in benefits etc, When I came to the UK, I thought this is absolutely the most brilliant system in the world. . .but then you discover that it often is not much more than a third world system’. (Leo: 610–615).

Leo’s disappointment and frustration were clear. He conveyed his feelings that although policies and systems are often in place to help, the reality became an illusion, being difficult to access.

Leo goes on to say:

‘I said, I’m coming from the same background, I had these sorts of issues, housing problems and set them down. But the thing is when I work. . . I try to understand the underlying issues for them, and you cannot say that when someone is sitting in front of you and they’ve got housing problems or money problems, they don’t have psychological problems, they come together’. (Leo: 85–92)

Jon highlights complex social rules embedded in the discussion of poverty.

‘You know, some clients get very upset especially if I bring in ‘money’ or speak with others about poverty or asking for money. . .it’s like a secret storm in my mind. . .’ (Jon: 271–273).

## Sub-theme – Isolation versus reaching out

Liv uses the image of a ladder in reaching out to show how poverty can lead to isolation and difficulties with focussing and counselling can help:

‘If you put a ladder in front of someone they will climb,. . .it’s when

being poor doesn’t allow people to focus and see where they are that’s

where counselling comes in to act and. . .small goals are important. . ..’ (Liv:

819–828).

Pete’s tone of voice during our conversation was gentle. It was his way of communicating the seriousness, the gravity of the current situation for himself and for others working in the field:

‘I would like clinical psychologists and counselling psychologists, and psychotherapists to learn about poverty and the social and political backgrounds of mental illness and psychological dysfunction, especially with a society that is becoming increasingly. . .unequal and which is getting bigger and bigger and can’t contain in mainstream therapy. . .’ (Pete: 541–550).

Pete’s request for understanding for recognition and understanding of a divided and unequal society as well as the multi-layered problems that present him and his clients with significant challenges. Issues around poverty and its resulting hardships, discrimination and shame are compounded by a lack of training, information and access to services, all of which warrant being included on training programmes as a matter of urgency. Pete’s observation that poverty has an impact on mental health by widening the divisions in society seemed timely in terms of offering therapy as a mainstream service when multi-cultural and multi-ethnic experiences need inclusion and greater understanding in order to support all members of society.

## Superordinate theme 3: Difficulties in navigating multiple challenges and barriers

This theme explores how participants managed these struggles by adopting various strategies or techniques, which helped them not only to prevent ‘burnout’ arising but to manage the isolating nature of their work. All participants in the study experienced obstacles such as accessing services to help their clients. Other obstacles the therapists experienced were a lack of training, adequate supervision and literature on issues relating to poverty. forming and maintaining boundaries when working with clients living in poverty, several seemed to struggle with this issue:

Three main sub-themes highlight this superordinate theme further:

1. Impact of support services and structures on advocacy
2. Averting Burnout: ‘I focus on self-care’
3. Redefining Professional Identity: Incorporating the unacknowledged Scientist Practitioner-Advocacy Model

## Sub-theme 1: Impact of support services and structures on advocacy

Borges (2014) found that organisations that support those living in poverty frequently do so without much support and without many resources, reporting that this can be likened to a parallel process which reflects the presenting problems of the very clients they serve.

Sam explained how he struggles to offer a reasonably prompt support to his clients who suffer with hardship:

‘. . .I don’t know how accessible IAPT services are for a lot of people. I’ve met a lot of clients who are on very long waiting lists and they just need help now. . . our waiting list is not that long, about three months maximum (Sam 161-164),. . .the government helps but this support is very limited’ (Sam 385).

Sam’s comments illustrate clearly how being poor has a negative impact for people who may be desperately in need of support, and whose lack of financial independence means being on a waiting list while people who can pay have immediate support.

Rose also voiced a complex mix of emotions in her work when she has to undertake work that might have been undertaken by other support services:

‘it’s frustrating to fill out a 40-page form to get Council Tax relief’ (Rose: 724).

And

‘where is the humanity in the decisions being made by government about austerity, you don’t earn £50,000 therefore you are of no value. So, it’s hard to then look at the whole person, their culture, social and how these so-called policy makers are grinding them down’ (Rose: 1113–1117).

Leo feels trapped in a loop:

‘yes, as I said, it’s like a loop, our staff are trying to help, but they’re struggling sometimes themselves, and it’s very difficult, really. I’m the manager here, but at the same time I’m a therapist myself, so I know the issues my staff are dealing with, they get frustrated with little pay’. (Leo: 525–32).

Rose and Leo’s accounts conveys a struggle in managing the harsh realities of working with clients and issues around poverty. Rose’s comment around how government policies impact her work was clearly evident in her transcript, as was her lack of confidence in her ability to support her clients living in poverty as a result. She expressed a certain degree of frustration with the work and its particular challenges. Leo’s comment about feeling ‘frustrated’ and in a ‘loop’, might suggest an unconscious identification with the experiences of his clients, and possibly, as a manager and as a therapist is well placed to understand a dynamic of struggling to live on a low income with little way out.

## Sub-theme 2: Averting Burnout: ‘I focus on self-care’

Therapists are often not adequately trained to consider issues of social inequality and may be ill prepared for working with clients living in poverty. This work can be demanding and requires active consideration, resilience and self care by those undertaking it. All of the participants shared an awareness that, by looking after themselves, they were better able to give to others, take control of their experiences, and actively manage the stress of advocating with limited resources. Kate expressed how self-care reduces burnout:

‘I focus on looking for burnout and manage myself. . .I join a band to relax. . .’ (Kate: 399–400*).*

Prior to her decision to find better ways to care for herself, Kate had suffered from a lot of turmoil and had struggled to cope with setting boundaries at work:

‘I feel isolated but need to support the client. . .’ (Kate 459–460).

Feeling isolated in the process of advocating for their clients and working with the broader social context, participants described how focusing on the clients’ goals, adapting their practice interchangeably with advocacy and therapy and empathising with clients’ experience helped with this obstacle. Participants felt they would have benefitted from being trained in modifying their treatment to better address transport and childcare needs as part of the therapy rather than acting solely as therapists. They felt this would have helped them be more effective as advocates, and to act as case managers and translators as well as to meet other requirements connected with their everyday experiences with their clients.

‘for me there’s a real sense of hopelessness, there’s a sense when do I retire’ (Rose: 831–832).

The systemic challenges were clearly part and parcel of a poverty trap, and being trapped, experienced by the clients. This was exacerbated through a decade of austerity measures and having to cope with a high cost of living on a low or sporadic income.

‘the effect of poverty,. . . it’s like a great big vice, a great big squeezer happening on the psyche, on the internal world. . . an emotional broken leg, punch to the chest, accident, freight train running over you, which leaves you in the same incapacity, that maybe having physical injuries from a car accident. . .the pressures of society, poverty being one, squeezes people out’ (Jon: 59–65).

Jon’s description of the impact of poverty offers some indication as to the gravity and complexity of therapists’ work with clients living in poverty. Although writing about psychiatry, the following statement may apply to anyone working within the area of mental health, ‘Inequality is a significant and reversible risk factor for mental disorders which demonstrates the essentially political nature of psychiatry’. Poole (2020 p. 191).

## Discussion

This study discussed the issues therapists reported facing in working with clients who are struggling with poverty related issues. The numbers are small but the findings indicate that further urgent work is needed as levels of poverty are rising in many countries. The major themes were the resilience and personal positioning of the therapists and their struggle to engage in this therapeutic work. In addition, issues relating to promoting social activism relating to poverty and navigating multiple challenges and barriers were the other two superordinate themes. The role of advocacy or social activism, by clinicians is a topic on which a range of opinions are held. Ignoring structural inequalities and the effects they may have on clients’ daily lives, may be to hide or collude with a narrative which ignores or minimises these, despite them forming a central aspect of the lived experience of some clients. Clinicians may at least, need to be familiar with other specialist services or resources which are available, and develop clear referral pathways to those who may be able to advocate on behalf of clients. The issue and positioning of social justice and associated issues within therapy appears to be one which requires further and urgent debate.

The reasons participants gave for engaging in this research included their desire to offer advocacy and therapy together, to effect change, while others felt that they were undertaking important work and wanted others to hear about this, these motivations will have influenced the result of the study. Feelings such as hopelessness, frustration, distress and dissatisfaction and the complex social rules that hide the discussion of poverty, all make it challenging to engage in this work. As does lack of training for many clinicians on working with people living with poverty. In addition, the difficult emotions associated with poverty and feelings related to class and ‘inferiority’, stigma and discrimination were all mentioned. Some of the participants noted the importance of their own personal and professional development, which included becoming more comfortable with their own power and identity, this was often accomplished through reflecting on class and their own positioning as well as reflecting upon the societal impact on those affected by poverty. A need for mental health training to create and encourage a safe forum where issues and experiences of social justice including but not limited to poverty, classism, differences, racism and oppression are recognised and can be discussed seems important if the principles of social justice are to be upheld and the needs of people living in poverty who attend therapy are to be recognised. More research is needed into poverty and mental health and the role of the clinician., how these difficulties may affect the work and how such difficulties may be managed. The lack of attention to this central and potentially life determining experience appears disappointing given the high numbers of individuals living in poverty and the projected rise in these numbers.
